# Primary Pleomorphic Adenoma of the External Auditory Canal: A Case Report and Review of the Literature

**DOI:** 10.1155/2014/975151

**Published:** 2014-04-07

**Authors:** Chizu Saito, Takeharu Kanazawa, Takehiko Yamaguchi, Ken-ichi Nakamura, Keiichi Ichimura

**Affiliations:** ^1^Department of Otolaryngology/Head and Neck Surgery, School of Medicine, Jichi Medical University, 3311-1 Yakushiji, Shimotsuke 329-0498, Japan; ^2^Department of Otolaryngology, Shin-Oyama City Hospital, 1-1-5 Wakagi-cho, Oyama 323-0028, Japan; ^3^Department of Pathology, School of Medicine, Jichi Medical University, 3311-1 Yakushiji, Shimotsuke 329-0498, Japan

## Abstract

*Background*. Pleomorphic adenoma (PA) is a benign tumour that mainly arises from salivary glands, and PA of the external auditory canal (EAC) is very rare. The objective of this study was to clarify the clinical presentation and treatment of PA of the EAC. *Method*. The authors present a case of PA arising from the EAC together with a literature review. *Results*. A 40-year-old man complained of hearing loss and foreign-body sensation of the right ear. Clinical and radiological examinations revealed a well-defined tumour limited to the EAC, with no connection to the parotid gland. Preoperative fine-needle aspiration cytology findings were characteristic of PA. The tumour was removed en bloc with the overlying skin. *Conclusion*. PA of the EAC is very rare, and methods to rule out malignancy before treatment are lacking. Thus, long-term follow-up is necessary, because malignant tumours are common in the EAC and PA has malignant potential.

## 1. Introduction

Pleomorphic adenoma (PA) is a benign tumour that mainly arises from the salivary glands [1]. However, PA may also arise from the external auditory canal (EAC), although reports are very rare. Since 1951, when Mark and Rothberg published their first EAC pleomorphic adenoma report [2], at least 35 similar cases have been reported [3–13]. PA of the EAC is classified as a type of ceruminal gland tumour. The ceruminal glands may give rise to both benign and malignant tumours. According to the World Health Organization (WHO) classification [14], the benign tumours include adenoma, pleomorphic adenoma, and syringocystadenoma papilliferum, and the malignant tumours include adenocarcinoma, adenoid cystic carcinoma, and mucoepidermoid carcinoma. Ceruminal tumours are frequently malignant with a poor prognosis and extend to the middle ear inducing significant hearing loss [8]. PA arising from the EAC is the rarest type of ceruminal gland tumour, and there is a scarcity of information regarding differentiation between PA and malignant tumours.

In this case report, we describe a rare finding of a PA arising from the EAC and review the literature on these tumours.

## 2. Case Report

**Figure 1:**
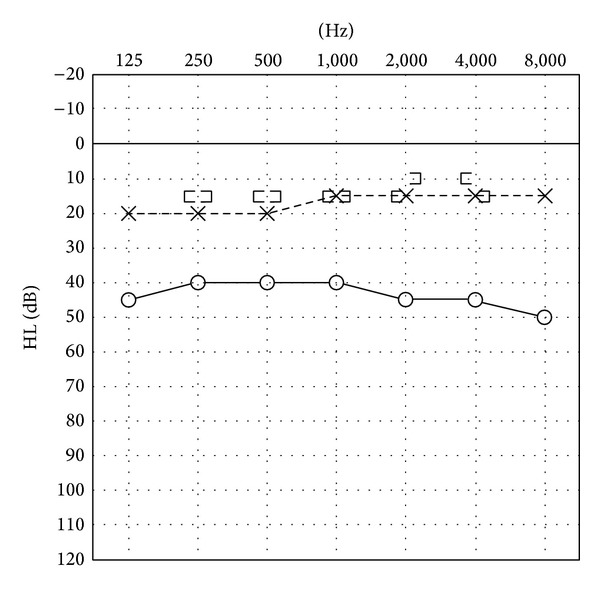
Preoperative pure-tone audiogram. A pure-tone audiogram revealed conductive hearing loss of 30 dB.

A 40-year-old man was admitted to our hospital with hearing loss and foreign-body sensation of the right ear that had been present for the previous 5-6 years. Ear discharge and pain were absent, but a tumour covered by normal skin was observed in the right EAC. A pure-tone audiogram revealed conductive hearing loss of 30 dB(Figure 1).

**Figure 2:**
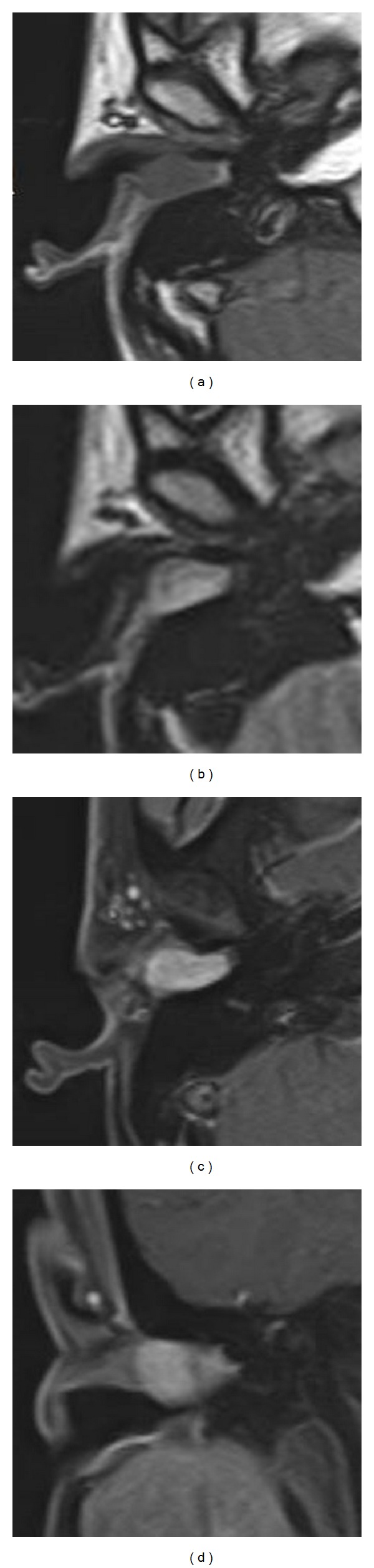
Magnetic resonance images. The tumour was detected as a 2.3 × 2.1 × 1.8 mm mass in the EAC, with low signal intensity on T1-weighted, high signal intensity on T2-weighted, and homogeneous enhancement on gadolinium-enhanced T1-weighted images. (a) Transverse section of a T1-weighted image. (b) Transverse section of a T2-weighted image. (c) Transverse section of a gadolinium-enhanced T1-weighted image. (d) Coronal section of a gadolinium-enhanced T1-weighted image.

A computed tomography (CT) scan showed that the soft tissue mass was confined to the right EAC, with no erosion of adjacent bone. On magnetic resonance imaging (MRI), the tumour was detected as a 2.3 × 2.1 × 1.8 mm mass on the posterosuperior wall of the EAC, with low signal intensity on T1-weighted, high signal intensity on T2-weighted, and homogeneous enhancement on gadolinium-enhanced T1-weighted images (Figure 2). There was no connection with the parotid gland and no invasion of the middle ear. Preoperative fine-needle aspiration cytology findings were typical of PA.

A retroauricular surgical approach was made to obtain good visualization of the tumour. The tumour was attached to the posterosuperior canal wall and was removed with overlying skin. After tumour removal, the tympanic membrane was observed to be intact, and no bone destruction was detected in the canal wall. A postoperative pure-tone audiogram revealed normal hearing. The patient remains free of recurrence at 12 months and continues to be followed up.

**Figure 3:**
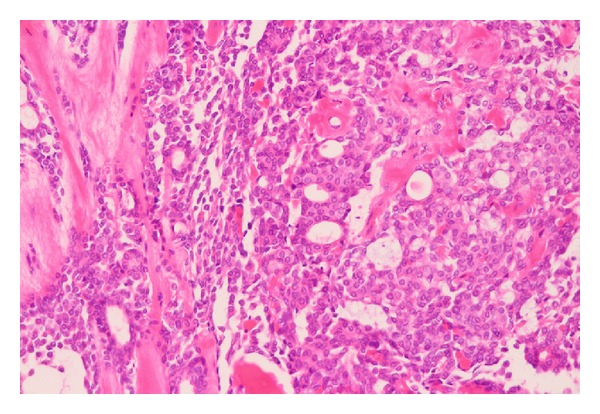
Histopathological section of the tumour. Histopathological examination of haematoxylin and eosin (HE) stained sections revealed a partly encapsulated tumour exhibiting proliferation of myoepithelial cells with foci of ductal differentiation associated with myxoid and hyalinized matrix material (×40).

Microscopically, histopathological examination of haematoxylin and eosin (HE) stained sections revealed a partly encapsulated tumour exhibiting proliferation of myoepithelial cells with foci of ductal differentiation associated with myxoid and hyalinized matrix material (Figure 3).

## 3. Discussion

Controversy exists regarding whether PA of the EAC originates from ceruminous or ectopic salivary gland tissue. However, at present, circumstantial evidence strongly suggests the majority of such tumours are of ceruminous gland origin [3]. According to the WHO classification [14], benign ceruminal gland tumours include adenoma, chondroid syringoma (pleomorphic adenoma), and syringocystadenoma papilliferum, and malignant ceruminal gland tumours include adenocarcinoma, adenoid cystic carcinoma, and mucoepidermoid carcinoma. Chondroid syringoma is similar to the PA of salivary glands [14]. On the other hand, Wetli et al. suggested that tumours of ceruminal gland origin should be classified into four categories: adenoma, pleomorphic adenoma, adenocarcinoma, and adenoid cystic carcinoma [15]. This widely accepted classification system clearly distinguishes two benign (ceruminous adenoma and pleomorphic adenoma) and two malignant (ceruminous adenocarcinoma and adenoid cystic carcinoma) categories. Regardless of which classification system is used, it is clear that malignant tumours commonly arise from the EAC and that PA of the EAC is relatively rare.

To date, Haraguchi and others have provided information on 35 published cases of primary PA of the EAC. The age at presentation ranged from 15 to 80 years, with a mean age of 49.7 years. There was no sex predilection. Common symptoms related to primary PA of the EAC included obstruction of the EAC meatus, hearing loss, otalgia, and otorrhoea [3–13].

With regard to the site in the meatus, the tumour derived from the posterior wall in 8 cases, posterosuperior wall in 7 cases, superior wall in 3 cases, anterior wall in 6 cases, and anteroinferior wall in 2 cases. The posterior and posterosuperior walls represented the most common sites, although the tumours originated from any location in the EAC. Furthermore, average size did not differ significantly among sites [3–13]. CT and MRI examination were found effective for preoperative diagnosis of PA. In general, CT revealed the tumours as well defined with no erosion of adjacent bone. MRI also revealed a well-defined margin, as well as hypointensity on T1-weighted images and hyper- to low intensity on T2-weighted images (Table 1) [4, 5, 7, 8]. The tumours were enhanced by contrast material. The tumour in this case was detected as mass on the posterosuperior wall of the EAC, with low signal intensity on T1-weighted, high signal intensity on T2-weighted, and homogeneous enhancement on gadolinium-enhanced T1-weighted images.

These MR features, in contrast to high-grade malignancies, are compatible with benign tumours or low-grade malignancies, similar to PA of the salivary glands, but they are not specific for a diagnosis of PA and cannot completely rule out malignancy.

Thus, pathological diagnosis is required to differentiate between benign and malignant tumours such as adenocarcinoma and adenoid cystic carcinoma. Although there is a report that fine-needle aspiration cytology contributes to preoperative diagnosis of PA [4], preoperative incisional biopsies were performed in most previously reported cases. In our case, preoperative fine-needle aspiration cytology findings were typical of PA.

The most recommended treatment is complete local excision with an adequate margin of normal tissue. The surgical approach for complete excision depends on size and extension of the tumour. The previously reported cases underwent mostly endaural incision and sometimes retroauricular incision, but bony canal excision or mastoidectomy was not commonly performed. Recurrences were reported in three cases, and one out of the 35 cases was malignant. These results suggest that complete excision and long-term follow-up are important in the management of PA of the EAC.

In our case, the tumour could be completely removed with overlying skin by using a retroauricular incision. However, long-term follow-up is necessary, because PA has the potential to recur or undergo malignant transformation.

## Figures and Tables

**Table 1:** Magnetic resonance imaging findings of reported cases.

	Present case	Masumura et al. [5]	Koyuncu et al. [7]	Tsukahara et al. [8]	Gerber et al. [4]
T1	Low	Low	—	Low	—
T2	High	High	Moderate	Low	—
Gd-enhanced	Homogenic-high	Homogenic-high	Homogenic	Homogenic	Moderate
